# Exposure to preference‐matched alcohol advertisements from national sports broadcasts increases short‐term alcohol consumption inclinations in risky drinkers

**DOI:** 10.1002/hpja.894

**Published:** 2024-07-01

**Authors:** Ross C. Hollett, Jesse Fairclough, Julia Butt, Brennen Mills

**Affiliations:** ^1^ Psychology and Criminology, Edith Cowan University Joondalup Western Australia Australia; ^2^ School of Medical and Health Sciences, Edith Cowan University Joondalup Western Australia Australia

**Keywords:** advertising, alcohol, craving, experiment, sports

## Abstract

**Background:**

In Australia, sports broadcasting is afforded special alcohol advertising rights during daytime hours, which raises public health concerns, including short‐term increases in alcohol consumption among the broad viewership of national sporting codes.

**Methods:**

We conducted a content analysis across a sample of nationally televised finals matches (*N* = 16) from the Australian Football League (AFL) and the National Rugby League (NRL) to determine the prevalence of alcohol advertising video clips during these broadcasts. We also conducted an online experiment exposing participants (*N* = 345) to a randomly selected alcohol advertisement and measured the immediate effects on self‐reported alcohol craving and drinking intentions.

**Results:**

The prevalence of alcohol advertising video clips during AFL broadcasts was 3.9% and 1.8% for NRL. While, overall, alcohol advertisement video clip exposure did not impact craving or drinking intentions, a modest increase in craving was found for a subsample of risky drinking participants (*N* = 107) who also reported a preference for the specific alcoholic beverage being advertised.

**Conclusions:**

Video alcohol advertisements occurred less than 1 in 20 advertisements on average and exposure to alcohol advertising elicited a low, yet measurable, short‐term increase in alcohol inclinations, among vulnerable adult drinkers when a desirable alcoholic beverage advertisement is viewed.

**So What?:**

Given that alcohol advertisements are most likely to increase consumption among risky drinkers, health messaging during sports broadcasts needs to specifically target these individuals.

## 1 INTRODUCTION

Concerns surround the prevalence of alcohol advertising during sports broadcasts and its potential contribution to harmful drinking patterns among viewers.^1^, ^2^, ^3^, ^4^, ^5^, ^6^, ^7^ In Australia, exemptions allow alcohol advertisements during national sports broadcasts at any time on weekends, while restrictions limit such advertisements before 8:30 PM on other days.^8^ The high viewership of national sports in Australia, such as 4 million viewers for the 2021 Australian Football League (AFL) grand final,^9^ illustrates the reach of alcohol advertisements during these events and their potential to increase consumption while undermining other health messaging efforts.^10^ Existing claims about the adverse health effects of alcohol advertising during sports broadcasts primarily rely on correlational designs and exposure paradigms that may not reflect the current sports broadcasting context.^1^, ^11^, ^12^ Given the adverse health consequences associated with alcohol consumption,^13^ it is imperative to investigate whether contemporary alcohol advertisements during far‐reaching sports broadcasts elevate the immediate risk of harmful drinking behaviour.

Extensive cue‐exposure literature demonstrates alcohol‐related imagery triggers heightened alcohol craving, intentions to drink, alcohol attentional biases and actual drinking behaviour.^14^, ^15^, ^16^, ^17^ Addiction models are useful for understanding the mechanisms, which underlie responsiveness to alcohol cues among social and high‐risk drinkers. For instance, the ambivalence model of alcohol craving assumes that inclinations to consume alcohol exist along two independent continuums of approach and avoidance.^18^ Simultaneously acknowledging motivations to approach alcohol and aversive motivations to avoid alcohol appropriately reflect the neuropsychological foundations of addiction and reward‐seeking behaviour.^19^ Specifically, when substance‐related cues activate reward circuitry, inhibitory functions become overwhelmed with the strengthening of bottom‐up (e.g., incentive sensitization) and weakening of top‐town processes.^20^, ^21^ Repeated alcohol use can heighten the reward salience and produce appetitive/inhibitive imbalances, which explains compulsive substance‐seeking behaviours among high‐risk drinkers despite the adverse consequences. Accordingly, high‐risk drinkers consistently show stronger responses to alcohol cues than low risk/social drinkers.^14^, ^22^, ^23^ However, experiments testing these theoretical frameworks often deviate from real‐world conditions, using researcher‐manufactured visual material in contrast to the brief high‐quality advertisements aired during sports breaks. The few experimental studies specifically examining alcohol advertising on drinking intentions and behaviours have yielded null or modest effects and are outdated.^24^, ^25^, ^26^, ^27^ Many systematic reviews on alcohol advertising rely on non‐experimental designs or conclude that experimental evidence of harm is inconsistent or unsubstantiated.^28^, ^29^, ^30^, ^31^ While one systematic review and meta‐analysis of experimental studies examining immediate drinking behaviours concluded that various types of alcohol advertising (print, film and television) exert modest increases in alcohol inclinations, these results were derived from a mixture of children, adolescent and adult samples under controlled laboratory conditions.^27^ In sum, previous research has not established a causal link between the brief alcohol‐related video advertising typically used in sports broadcasts and immediate inclinations to drink alcohol. While the scope of the present study is limited to understanding the immediate effects of alcohol advertising, we assume that an accumulation of such effects might contribute to longer term changes in alcohol‐related attitudes and behaviours. To guide current policy, contemporary experimental evidence is needed to understand both the prevalence and potential harm of alcohol advertising within national sporting broadcasts.

The present study seeks to (1) quantify the proportion of video alcohol advertising during high‐exposure sports broadcasts and (2) provide experimental evidence of its influence on alcohol drinkers' immediate inclinations to consume alcohol. We also explored several factors, which heighten the risk that alcohol advertising will increase craving and intentions to drink. First, as alcohol consumption is generally more popular in the afternoon/evening (PM) than in the morning (AM),^32^ and high‐profile sports broadcasts generally occur in the afternoon/evening local time,^33^, ^34^ we included AM/PM as a quasi‐experimental factor. Second, alcohol advertisements often utilise positively valanced alcohol contexts (people socialising in pubs/bars) to facilitate generalised drinking inclinations,^35^ but we assumed that advertisements featuring a preferred alcoholic beverage are more likely to elicit an appetitive response. Accordingly, we examined whether effects were stronger when participants were exposed to one of their preferred alcoholic beverages. Finally, factors assumed to strengthen responses to alcohol cues, such as historical risky alcohol use, higher levels of sensation seeking and impulsivity, were also measured to explore whether they moderate appetitive motivational responses.^14^, ^16^, ^36^ The risky alcohol use measure was also used to classify low and risky drinking groups so we could include this as a quasi‐experimental factor. Consistent with cue‐exposure literature and our assumptions above, the following hypotheses were tested:

(a) Alcohol craving and (b) intentions to drink would increase following exposure to alcohol advertising, but these effects were expected to be (c) stronger for those exposed in the afternoon/evening (PM) compared to those exposed in the morning (AM).
(a) Alcohol craving and (b) intentions to drink would increase following exposure to alcohol advertising among those who were exposed to an advertisement matching their alcoholic beverage preferences, but these effects were expected to be (c) stronger for those exposed in the afternoon/evening (PM) compared to those exposed in the morning (AM).
For both experimental analyses, risky drinkers were expected to show larger increases in their (a) craving and (b) intentions to drink relative to low‐risk drinkers.
Trait‐level (a) alcohol use risk, (b) sensation seeking and (c) impulsivity measures would positively correlate more strongly with post‐advertisement exposure alcohol craving and intentions to drink than pre‐advertisement exposure ratings.

## 2 METHOD

### 2.1 Participants

Australian adult alcohol drinkers (*N* = 346) were recruited from an undergraduate course credit scheme (54%), the general community via a sponsored social media post (16%) and via a Qualtrics panel (30%).^i^ There were similar proportions of men (48%) and women (49%) and a few not identifying as either (3%). Participants were aged between 18 and 80 years (M = 37.44, SD = 16.78) and were mostly Caucasian (82%), followed by Asian (8%), Aboriginal or Torres Strait islander (3%), African (1.5%), or other (5.5%). Almost half (48%) of participants reported watching either AFL or National Rugby League (NRL) regularly (once a month or more), and 70% of participants viewed professional sporting broadcasts at least once a month.

### 2.2 Materials

#### 2.2.1 Alcohol craving and intention to drink

Alcohol craving was conceptualised according to the Ambivalence Model,^18^ whereby approach (inclinations to consume alcohol) and avoidance (inclinations to not consume alcohol) are assumed to fluctuate independently and were measured separately. Consistent with prior experimental research on alcohol video exposure,^14^ we used separate single items for approach and avoidance, each rated on an 8‐point Likert scale (1—very weak to 8—very strong). Specifically, “Often people who drink alcohol have two voices in their head: One that says ‘I really want a drink right now’ (approach), and another that says ‘I really *do not* want a drink right now’ (avoidance) and the two statements were rated separately via an 8‐point Likert scale. Avoidance ratings were then subtracted from approach ratings to create a single composite score. Drinking intentions in response to the video advertisement were measured using the item “I intend to drink alcohol as soon as I get the chance”, rated from 1 (strongly disagree) to 5 (strongly agree).

#### 2.2.2 Alcohol use risk

The 10‐item Alcohol Use Disorder Inventory (AUDIT^37^) was used to screen out non‐drinkers (item 1) and estimate risky use of alcohol. Item responses (scored from 1 to 4) were summed to calculate a total score. Total scores of 1–7 indicate low‐risk alcohol use, and scores of 8+ indicate risky alcohol use.^38^ These criteria divided participants into low‐risk (*N* = 189) and risky (*N* = 157) drinking groups to test the potential moderating effects of prior alcohol use on experimental variables. The AUDIT demonstrated good internal consistency (α = 0.86).

#### 2.2.3 Brief sensation‐seeking scale

The eight‐item Brief Sensation Seeking Scale (BSSS^39^) was used to estimate individual differences in sensation‐seeking propensity. Responses were made on a 5‐point Likert scale, anchored from (1) strongly disagree to (5) strongly agree. Items responses were averaged to calculate a total score. The BSSS demonstrated good internal consistency (α = 0.78).

#### 2.2.4 Impulsivity

The brief Barrat Impulsivity Scale (BIS^40^) was used to estimate individual differences in impulsiveness. Responses were made on a 4‐point scale anchored from (1) Rarely/Never to (4) Almost Always/Always. Item responses were averaged to calculate a total score. The BIS demonstrated good internal consistency (α = 0.81).

#### 2.2.5 Sports viewership habits

Participants selected which professional sports they watched at least once per month (from 30 categories). Participants also selected whether they used free‐to‐air or subscription to access professional sporting broadcasts (either exclusively or a mixture of both).

#### 2.2.6 Alcohol advertisements

Across the final month of the 2022 AFL and NRL seasons, video recordings of eight matches were obtained (four AFL and four NRL) across subscription and free‐to‐air services, totalling 16 broadcasts (including grand finals). Two independent raters were randomly allocated eight broadcasts to count the occurrence of video alcohol advertisements. Only video advertisements occurring during dedicated commercial/scoring breaks were counted. Two matches (one free‐to‐air and one subscription) were randomly selected to be cross‐coded for inter‐rater reliability which was estimated using an intra‐class correlation coefficient and found to be excellent (0.77–0.96).^41^ Following content analysis, all the identified alcohol advertisements were edited into isolated clips of approximately 30 s and privately hosted on YouTube to facilitate experimental exposure. There were five unique alcohol advertisements in total, including two different Great Northern (beer) advertisements, two different Jameson (premixed whiskey) advertisements and one Jim Beam (premixed whiskey) advertisement. Descriptions of the alcohol advertisements and links to the Youtube videos can be found in the supplementary materials (Supporting Information S1 and S2).

### 2.3 Procedure

The Qualtrics survey platform^42^ deployed the experiment. Initially, participants provided consent and completed baseline pre‐exposure measures of craving and drinking intentions. Participants were then randomly allocated to view one of the five alcohol videos. Participants (*N* = 29) taking longer than 60 s to progress from the commencement of the video to the commencement of the post‐exposure ratings of craving and drinking intention were excluded to mitigate the potential impact of distraction. We also excluded an additional 51 cases for failing a self‐report attention check. After post‐exposure measures, participants completed the AUDIT, BSSS and BIS in randomised order, followed by sports viewership, alcoholic beverage preferences and demographic questions. University‐recruited participants were awarded course credit, social media participants entered a prize draw and Qualtrics panel participants received points, gift cards or monetary remuneration depending on panel arrangements. The research was approved by the university human research ethics committee.

### 2.4 Research design and analysis

This study used both experimental and correlational designs. For the experimental design, two separate mixed‐model ANOVAs were used to test the effects of three independent variables, a within‐subjects factor (Alcohol Exposure: Pre; Post) and two between‐subjects factors (exposure time: AM and PM, and AUDIT group: low risk and risky), on the dependent variables of craving and drinking intentions. These analyses were repeated for a subsample of participants exposed to an advertisement, which matched their alcoholic beverage preferences. For the correlational design, Pearson correlations were performed between experimental measures (craving; drinking intentions) and trait measures (AUDIT, BSSS and BIS).

## 3 RESULTS

### 3.1 Content analysis

A total of 774 video advertisements were identified across the 16 broadcasts, of which 24 were for alcohol (3.1%). The proportion of alcohol advertisements was higher for AFL (3.9%) compared to NRL (1.8%) and higher for subscription (3.7%) compared with free‐to‐air services (2.8%).

### 3.2 Experimental results for craving and drinking intentions

For the craving score, there was no main effect of alcohol exposure, *F*(1, 342) = 2.04, *p* = 0.154, η_p_^2^ = 0.01, and no two‐ or three‐way interactions (*p*s > 0.178). However, there was a main effect of exposure time, *F*(1, 342) = 9.00, *p* = 0.003, η_p_^2^ = 0.03 and AUDIT group, *F*(1, 342) = 44.55, *p* < 0.001, η_p_^2^ = 0.112. Specifically craving scores at both pre‐exposure (*p* = 0.036, *d* = −0.23) and post‐exposure (*p* = 0.006, *d* = −0.30) were higher for those who were exposed to advertisements in the afternoon/evening, compared to those in the morning (see Table 1.). Furthermore, craving scores at both pre‐exposure (*p* < 0.001, *d* = −0.64) and post‐exposure (*p* < 0.001, *d* = −0.71) were higher for those who were classified as risky drinkers, compared to those classified as low risk drinkers.

**TABLE 1:** Descriptive statistics and correlations between experimental and trait measures.

Experimental	Correlations			Descriptives	
	Trait				
	AUDIT	BSSS	BIS	Mean	SD
AM (*N* = 149)					
Craving pre	**0.47**	0.06	**0.19**	−3.18	3.97
Craving post	**0.47**	0.12	0.13	−3.17	4.01
Intentions pre	**0.46**	0.14	0.15	2.28	1.09
Intentions post	**0.47**	0.14	**0.17**	2.20	1.11
PM (*N* = 196)					
Craving pre	**0.38**	**0.18**	**0.27**	−2.24	4.05
Craving post	**0.41**	**0.20**	**0.33**	−1.92	4.19
Intentions pre	**0.48**	**0.29**	**0.27**	2.30	1.13
Intentions post	**0.52**	**0.32**	**0.28**	2.35	1.17
Mean	8.61	2.79	2.11		
SD	6.70	0.81	0.52		

*Note*: Significant (*p* < 0.05) correlations in boldface.

Abbreviations: AUDIT, Alcohol Use Disorder Inventory Test; BIS, Barratt Impulsivity Scale (brief); BSSS, Brief Sensation Seeking Scale.

For drinking intentions, there was no main effect of alcohol exposure *F*(1, 342) = 0.06, *p* = 0.811, η_p_^2^ = 0.00, exposure time, *F*(1, 342) = 1.38, *p* = 0.241, η_p_^2^ = 0.00, and no two‐ or three‐way interactions (*p*s > 0.071) indicating the alcohol advertisements had no measurable effect on drinking intentions in the morning or the afternoon/evening (see Table 1.). However, there was a main effect of AUDIT group such that drinking intentions at both pre‐exposure (*p* < 0.001, *d* = −0.71) and post‐exposure (*p* < 0.001, *d* = −0.80) were higher for risky drinkers, compared with low‐risk drinkers. Given that neither craving or drinking intentions significantly increased, hypotheses 1a, 1b and 1c were not supported.

**FIGURE 1:**
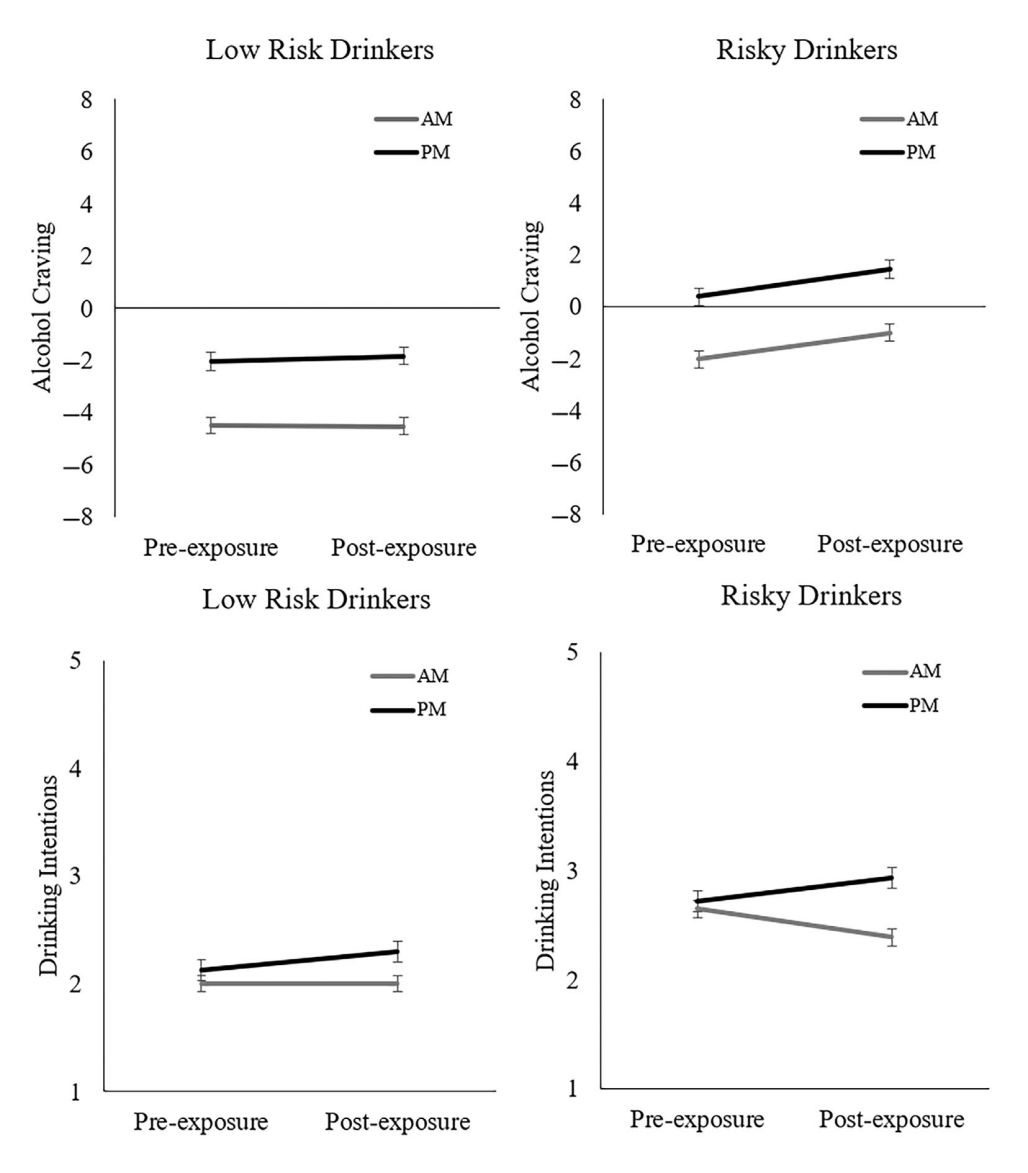
Means and 95% confidence intervals for craving and drinking intentions in risky and low‐risk drinking participants who were exposed to an advertisement which matched their alcoholic beverage preferences, separated for AM and PM exposure times.

The same analyses were then performed for those who were exposed to an alcohol advertisement, which matched at least one of their alcoholic beverage preferences (*N* = 107). For the craving score, there were main effects of alcohol exposure, *F*(1, 103) = 5.70, *p* = 0.019, η_p_^2^ = 0.05, exposure time, *F*(1, 103) = 13.31, *p* < 0.001, η_p_^2^ = 0.11, AUDIT groups, *F*(1, 103) = 18.05, *p* < 0.001, η_p_^2^ = 0.15, and a two‐way interaction between alcohol exposure and AUDIT groups, *F*(1, 103) = 4.21, *p* = 0.043, η_p_^2^ = 0.04. No other two‐ or three‐way interactions were significant (*p*s > 0.78). Specifically, there was a significant increase in craving from pre‐exposure (*M* = −1.95, *SD* = 4.00) to post‐exposure (M = −1.39, SD = 4.32) across both AM and PM groups (*p* = 0.02, *d* = −0.23), which partially supports hypothesis 2a. Note that the means at pre‐exposure (*p* = 0.001, *d* = −0.64) and post‐exposure (*p* = 0.002, *d* = −0.63) were significantly larger for the PM group compared to the AM group (see Figure 1). Importantly, and in support of hypothesis 3, when analysing AUDIT groups separately, risky drinkers showed a significant increase (*p* < 0.001, *d* = −0.60) in craving following post‐exposure, whereas low‐risk drinkers did not (*p* = 0.818, *d* = −0.03).

For drinking intentions, there were no main effects of alcohol exposure, *F*(1, 103) = 0.17, *p* = 0.682, η_p_^2^ = 0.00, or exposure time, *F*(1, 103) = 1.73, *p* = 0.191, η_p_^2^ = 0.02. However, there was a main effect of AUDIT group, *F*(1, 103) = 8.15, *p* = 0.005, η_p_^2^ = 0.07, and an interaction between alcohol exposure and exposure time, *F*(1, 103) = 5.34, *p* = 0.023, η_p_^2^ = 0.05. Follow‐up tests revealed those exposed to in the morning showed a *decrease* in drinking intentions from pre‐ to post‐exposure (*p* = 0.159, *d* = 0.21), and those who were exposed in the afternoon/evening showed an *increase* in drinking intentions from pre‐ to post‐exposure (*p* = 0.070, *d* = −0.24), neither change was significant. Therefore, hypotheses 2b and 2c were not supported. Drinking intentions were higher at post‐exposure for the PM group compared to the AM group (*p* = 0.05, *d* = −0.38), but this difference was equal to the threshold for significance. See Figure 1 for means and confidence intervals.

### 3.3 Correlational analysis with individual differences measures

As can be seen in Table 1, all PM correlations were significant, and the strongest associations were between the experimental variables and the AUDIT. The strength of all associations was similar at pre‐ and post‐exposure (confirmed via Fisher's Z comparisons, *p*s > 0.26), suggesting that higher AUDIT, BSSS and BIS scores did not correspond with greater risk of increased craving or drinking intentions following alcohol exposure compared to baseline. Therefore, hypotheses 4a, 4b and 4c were not supported.

## 4 DISCUSSION

The present study offers insight into the prevalence of alcohol advertising in contemporary sports broadcasts and the potential effects on viewer inclinations to consume alcohol. We found video alcohol advertising was not highly prevalent in two national sports during the highest viewership period of the season. With 24 alcohol video advertisements identified across 16 matches, this corresponded to around one to two advertisements per match. Prior studies including a wider range of alcohol advertisements in their content analyses (e.g., presence of logo/banners), reported higher prevalence estimates (10%) during high‐profile AFL broadcasts.^43^ Given the present study was unable to demonstrate an impact of a 30 s alcohol advertisement exposure on craving or drinking intentions when the advertisement is randomly selected, we conclude that 30‐s advertisements are unlikely to greatly increase viewers' immediate drinking inclinations. However, we assume these brief videos are still an expensive investment by brands in the hope of measurable longer‐term returns in product awareness and sales even if they do not produce immediate effects. There are also the effects of peripheral material embedded within commentary, banners and product placement, which may contribute to the cumulative effects of alcohol advertising throughout a match or season. While it is challenging to experimentally determine the impact of all these forms of advertising on alcohol consumption inclinations, we encourage future researchers to consider the relative impact of these various forms of exposure, particularly on sociocultural attitudes towards alcohol.

When analysing participants who reported a preference for the beverage depicted in the advertisement, there was a modest overall increase in craving but not drinking intentions. Importantly, drinking risk history moderated craving responses in the beverage‐matched sample, such that risky drinkers reported stronger increases in craving relative to low‐risk drinkers. Results confirmed assumptions that craving and drinking intentions are generally stronger in the afternoon/evening than the morning.^32^ Given the typical afternoon/evening programming of AFL and NRL matches, we regard data from the PM group to be more externally valid in this context.

The present findings align with prior research suggesting exposure to video alcohol advertisements produces only negligible or modest effects,^24^, ^25^, ^26^ and these effects are stronger for those who report riskier prior alcohol use.^14^ While laboratory cue‐exposure studies have yielded stronger effects, these studies occur in highly controlled settings (e.g.,^14^). However, sports alcohol advertising exposure does not occur under controlled conditions and sports audiences may be concurrently socialising, consuming food and generally distracted by aspects of their environment. Importantly, our findings indicate viewers are more motivated to drink alcohol when advertisements feature a preferred beverage. Given that 31% of our sample reported a beverage preference match when alcohol advertisements were randomly assigned, this could represent a significant number of viewers who are vulnerable to increases in their inclination to drink alcohol. It might also be valuable in further research to explore whether participants identify with the actors and contexts depicted in the advertisements as this may enhance the positive valence elicited by an advertisement.

While the findings may not support further restrictions to alcohol video advertising during high‐profile sports broadcasts, we only explored short‐term inclinations in adults. Our data do not contribute to understanding of longer‐term effects of alcohol advertisements on alcohol consumption and subjective norms, particularly in young people, which are important considerations with respect to advertising policies. Given sports broadcasting is widely viewed across age groups^44^ and AFL and NRL matches air for around eight continuous months each year, the capacity for longer term effects of alcohol advertisements during sports broadcasts on harmful alcohol use cannot be disputed. Further experimental research should be conducted, particularly with adolescents and longitudinally (e.g., across an entire season) to determine whether contemporary sports alcohol advertising increases alcohol expectancies, normalisation of alcohol use and harmful drinking.

## 5 CONCLUSIONS

Overall, a single exposure to an alcohol advertisement is unlikely to elicit an immediate motivational profile among adult drinkers that would likely trigger harmful drinking. However, our data suggest audiences are at greater risk of elevated craving and drinking intentions during afternoon/evening broadcasts, if they are a risky drinker, and if they are exposed to an advertisement containing a preferred alcoholic beverage.

## CONFLICT OF INTEREST STATEMENT

The authors declare no conflicts of interest.

## ETHICS STATEMENT

The data collection procedures reported in this manuscript were approved by the Edith Cowan University Ethics Committee.

## Supporting information


**Data S1.** Supporting Information.

**Data S2.** Supporting Information.

## Data Availability

The data that support the findings of this study are openly available in Open Science Framework at https://osf.io/twq9h, reference number DOI 10.17605/OSF.IO/2AN4B.

## Endnote

^i^
Qualtrics is a market research service provider: https://www.qualtrics.com/au/research-services/online-sample/.
