# Supplementary material for: Exposure to preference‐matched alcohol advertisements from national sports broadcasts increases short‐term alcohol consumption inclinations in risky drinkers
Source: Health Promot J Austr. 2024 Jul 1;36(1):e894. doi: 10.1002/hpja.894 (PMC11730253; doi:10.1002/hpja.894)
Supplement: Supplementary file 1 — Data S1. Supporting Information. [file HPJA-36-0-s001.docx]

| **Video** | **Description** |
| --- | --- |
| Great Northern Beer 1 | Close up on a fish cooking on an outdoor grill while white male (30-40s) squeezes lemon on it. Frame widens in one shot to show him retrieving fish from the grill with tongs in a remote coastal location and placing it on serving board. He walks past a tent with the serving board while another similar man walking towards him beer stubby in hand. An out of focus woman closes up tent in background. Man with stubby says “We on?”.  *Soft acoustic guitar and male vocals commence (“Time to escape coz I’m in need of warmer weather…)*  First man places fish and serving board next to a cooler full of Great Northern Beer. Unidentified hand reaches into the shot to pull out two beers from the cooler and hands one to the first man. First man opens the beer and takes a swig while the sunlight shines through the bottle. Final zoom out shot of campsite with three variations of the beer displayed in bottom right of screen. Deep male voiceover “Great Northern Brewing Co, the beer for up here”, text appears over the screen “THE BEER for UP HERE”. Fades to black. |
| Great Northern Beer 2 | Close up profile shot on woman’s head of dark-skinned woman age 30-40. Male voiceover: “You know, its got to make you wonder, how many stars would a hotel get…”. Woman smiles and emerges from a tent while camera pans to reveal green coastal value with ocean in distance during sunrise/sunset. Male voiceover: “…if every room had a view”. Upbeat acoustic guitar begins. Woman walks away from view and towards a beer being offered by male off screen, she says “cheers mate” and takes a swing with view in the background while light shining through the bottle.  *Male vocals commences (“Time to escape coz I’m in need of warmer weather…)*  Woman walks up to a campsite with two white males (both aged 30-40). One man passes a beer to another man. Cuts to a zoom out aerial shot of the campsite near the edge of a rocky outcrop with same coastal view visible in distance with three variations of the beer displayed in bottom right of screen. Deep male voiceover “Great Northern Brewing Co, the beer for up here”, text appears over the screen “THE BEER for UP HERE”. Fades to black. |
| Jameson 1 | Close up torso shot of bearded, long-haired white man age around 30 holding a red AFL ball. Electric guitar jamming background music. Cuts to woman and man in their 20s drinking a can of Jameson, soda, ginger and lime and glass filled with ice and same beverage (presumably) sitting at a table in backyard (first man with football in background about to kick it). Ball I kicked over the fence, cuts to a low shot of an olive-skinned woman in 20s wearing overalls while gardening as the ball lands in her arms. She smiles. Close up shot of unidentified hands leaning over a wooden fence opening a can of Jameson, soda, ginger and lime. Cuts back to gardening woman kicking the football back over the fence while laughing. She climbs over fence and original man with football shown kicking again. Ball bounces on rusting corrugated iron roof and lands in front of early 20s white man playing electric guitar in a basic dark brick mancave type setting (surfboards, couch, Jameson neon sign in background). Reveals that he is playing the background music. Music stops while he picks up the ball, there is a whistle off screen, he nods and smiles directed offscreen with football in his hands.  Cuts to shot of the man previously sitting at the outdoor table in a red Hawaiian style short, long hair and beard, slightly overweight. He handballs the football offscreen. More shots of the football being kicked. Male vocals begin *“Listen…(the rest is hard to decipher)”.*  Another young white man with a cap climbs over the fence and waves off screen. Young Asian woman walks along narrow fence way carrying Jameson 6-pack, man following close behind at dusk. Cuts to a party scene with hands in the air (now nighttime) with same characters as before smiling and drinking cans of Jameson while guitar man plays. (vague vocal music and guitar continues in the background)  Male voiceover “Jameson, Soda, Ginger and Lime, now available in a can”, text appears over the screen JAMESON, SODA, GINGER & LIME next to a close up of a Jameson spirit bottle, premix can and premix in a glass full of ice on a wooden board with two slices of lime next to the spirit bottle. Fades to black. |
| Jameson 2 | Same as above except depicting premixed cans with raw cola rather than soda and lime. |
| Jim Beam | Slow low zoom at street level towards a quiet town main street with old (1980s) cars parked near curb. Unclear person in the middle of the shot sweeping the sidewalk outside a cream building with a “The Wild Colt” sign. Male voiceover starts “We always believed the welcoming spirit…” cuts to bearded white man (in 30s) on the sidewalk with a neon “cocktails” sign above him while holding a broom speaking to the camera. He turns to face street to wave at someone then speaks to camera “…was a small-town thing”. Cuts to him briskly walking down the side of the building and continuing to speak “…turns out, we were wrong”. Soft upbeat music starts and fades into a nighttime street scene of same man entering an alleyway after opening a door. Voiceover continues “From the places that don’t have a welcome sign…”. Cuts to a close-up of the same man pausing as he enters a bar. Dark skinned woman passes a large beverage glass to the incoming man, they raise their glasses and smile (can hear barely audible man saying “welcome”). Camera pans over to new scene with a male sports team standing together in front of a bar, original man enters looking apprehensive, voiceover continues “…to the spots where you don’t look the part”. Sports team suddenly cheer at him while holding large glasses of beverage while man smiles and walks toward them/camera while getting pats on the back. Original man continues walking through a door with privacy glass out into a rocky sandy daytime location. His voiceover continues “…to moments where you don’t even know where you are”. He approaches a group of four at a campsite drinking and holding their glasses up, following a close up of an Asian woman raising her glass to him. He continues walking past them back to the original small town street setting from the start, his voiceover continues “…turns out the world can be a lot more welcoming than you think.” Cuts to empty bar scene then a close-up of a barman pouring liquid into a Jim Beam branded glass with ice and adding a slice of lime to rim. Last shot is a full bottle of Jeam Beam Bourbon on the bar next to the fully poured glass and the original man out of focus in the background seemingly waiting for a drink. Large texts appears “JIM BEAM (logo underneath), ALWAYS WELCOME”. |
