# Supplementary material for: Exposure to preference‐matched alcohol advertisements from national sports broadcasts increases short‐term alcohol consumption inclinations in risky drinkers
Source: Health Promot J Austr. 2024 Jul 1;36(1):e894. doi: 10.1002/hpja.894 (PMC11730253; doi:10.1002/hpja.894)
Supplement: Supplementary file 2 — Data S2. Supporting Information. [file HPJA-36-0-s002.docx]

Links to alcohol advertisements used as stimuli:

<https://youtu.be/ZwLXh2lj0NI>

<https://youtu.be/x0xZtcqUyQo>

<https://youtu.be/mxLODKZTHkA>

<https://youtu.be/phULJcFK0AQ>

<https://youtu.be/1U0xwcRa-1Q>
